# A physiologically based pharmacokinetic/pharmacodynamic model to determine dosage regimens and withdrawal intervals of aditoprim against *Streptococcus suis*

**DOI:** 10.3389/fphar.2024.1378034

**Published:** 2024-04-17

**Authors:** Kun Mi, Lei Sun, Lan Zhang, Aoran Tang, Xiaoyuan Tian, Yixuan Hou, Lingling Sun, Lingli Huang

**Affiliations:** ^1^ MOA Laboratory for Risk Assessment of Quality and Safety of Livestock and Poultry Products, Huazhong Agricultural University, Wuhan, China; ^2^ National Reference Laboratory of Veterinary Drug Residues (HZAU) and MOA Key Laboratory for Detection of Veterinary Drug Residues, Huazhong Agricultural University, Wuhan, China; ^3^ Department of Veterinary Medicine Science, College of Veterinary Medicine, Huazhong Agricultural University, Wuhan, China

**Keywords:** PBPK model, semi-mechanistic PD model, aditoprim, *Streptococcus suis*, dosage regimen, withdrawal interval

## Abstract

**Introduction:** Streptococcus suis (*S. suis*) is a zoonotic pathogen threatening public health. Aditoprim (ADP), a novel veterinary medicine, exhibits an antibacterial effect against *S. suis*. In this study, a physiologically based pharmacokinetic/pharmacodynamic (PBPK/PD) model was used to determine the dosage regimens of ADP against *S. suis* and withdrawal intervals.

**Methods:** The PBPK model of ADP injection can predict drug concentrations in plasma, liver, kidney, muscle, and fat. A semi-mechanistic pharmacodynamic (PD) model, including susceptible subpopulation and resistant subpopulation, is successfully developed by a nonlinear mixed-effect model to evaluate antibacterial effects. An integrated PBPK/PD model is conducted to predict the time-course of bacterial count change and resistance development under different ADP dosages.

**Results:** ADP injection, administrated at 20 mg/kg with 12 intervals for 3 consecutive days, can exert an excellent antibacterial effect while avoiding resistance emergence. The withdrawal interval at the recommended dosage regimen is determined as 18 days to ensure food safety.

**Discussion:** This study suggests that the PBPK/PD model can be applied as an effective tool for the antibacterial effect and safety evaluation of novel veterinary drugs.

## 1 Introduction

Aditoprim {ADP, 5-[(4-dimethylamino-3,5-dimethoxy-phenyl)methyl] pyrimidine -2,4-diamine}, belonging to diaminopyrimidines, can perform excellent antibacterial effects on Gram-positive and Gram-negative pathogens of swines (Cheng et al., 2017; Wang et al., 2022). The antimicrobial mechanism of ADP is the same as its structural analogs, such as trimethoprim (TMP) and diaveridine (DVD), which can block the folic acid synthesis of sensitive bacteria, inhibit bacterial growth, and enhance the antibacterial spectrum and antibacterial activity of sulfonamide drugs (Gleckman et al., 1981). ADP has demonstrated efficacy against *S. suis* (Cheng et al., 2017), a pivotal zoonotic pathogen in China, which has resulted in bacterial meningitis affecting thousands of humans (Feng et al., 2010). An empirical pharmacokinetic/pharmacodynamic (PK/PD) model of ADP against *S. suis* has been developed for the preclinical application (Qu et al., 2022). Nevertheless, limitations in the empirical PK/PD model have hindered its precision in designing dosage regimens.

(1) The empirical PK model, such as the compartmental model, represents the body as a system of one or more virtual compartments that do not correspond to the physiological and anatomical mechanisms (Fan and de Lannoy, 2014). It cannot extrapolate dose and simulate concentration–time curves in different tissues.
(2) PK/PD parameters played important roles in the determination of dosage regimens in veterinary medicine. The ratio of the area under the curve of plasma concentration to minimum inhibitory concentration (AUC/MIC) and the proportion of time that plasma concentration exceeds the MIC over the dosing interval (T > MIC) are routinely used in the empirical PK/PD model (Toutain et al., 2021). They highly rely on MIC. Measured error and subjective error could induce substantial uncertainty and variability in MIC, and the PK/PD parameter value would also be influenced (Nielsen et al., 2007). Additionally, PK/PD parameters only reflect a point estimate of the effect that ignores the time courses of drug concentration and antibacterial effect.
(3) Lacked evaluation of the safety. The empirical PK/PD model is not available for the assessment of antimicrobial resistance and drug residues.

A physiologically based pharmacokinetic (PBPK) model is characterized by anatomical and biochemical factors, which is a mechanistic approach to simulate the absorption, distribution, metabolism, and elimination of chemicals in the body. It has been applied for dose optimization (Zhou et al., 2021; Mi et al., 2022), food safety assessment of animal-derived food products (Lin et al., 2016; Henri et al., 2017), and chemical risk assessment (Chou and Lin, 2019). The semi-mechanistic PD model can quantify resistance development and bacterial regrowth under drug exposure. The PBPK/PD model, integrated PBPK model, and semi-mechanistic PD model can overcome the shortcomings of the empirical PK/PD model. By sets of mathematical equations, the PBPK/PD model can capture the time course of bacterial count under drug exposure and weaken the influence of MIC and PK/PD parameters. It can simulate the time course of bacterial count change and drug concentration.

The objective of this study is to establish a PBPK/PD model to describe the time course of bacterial count change under drug exposure and determine the preclinical dosage regimen of ADP against *S. suis*. A populational PBPK model was developed to determine the withdrawal intervals of the preclinical dosages to avoid residue violations and ensure food safety.

## 2 Materials and methods

### 2.1 Data source for PBPK model calibration

The physiological parameters (organ volumes and blood flow rates) of 25 kg of pigs were from the reported literature (Upton, 2008). The pharmacokinetic data used in the calibration and validation of the PBPK model are summarized in Table 1. Two different datasets were used to perform model calibration and validation. The graphic pharmacokinetic data were extracted from selected studies using WebPlotDigitizer (version 3.10, https://automeris.io/WebPlotDigitizer/).

**TABLE 1:** Data on ADP pharmacokinetic studies used for the calibration and validation of the PBPK model.

PK study/purpose	Route	Animal number	Dose regimen	Dose	Tissue
Calibration					
Wang et al. (2022)	IM^a^	6	Single injection	5 mg/kg	Plasma
(Wang, 2020)^b^	IM	40	12-h interval 14 doses	10 mg/kg	Liver, kidney, muscle, and fat
Validation					
Qu et al. (2022)	IM	6	Single injection	5 mg/kg	Plasma
(Wang, 2016)^b^	IM	40	24-h interval 7 doses	5 mg/kg	Liver, kidney, muscle, and fat

^a^ IM means intramuscular injection.

^b^ The data can be found in the Supplementary Material.

### 2.2 Model structure

**FIGURE 1:**
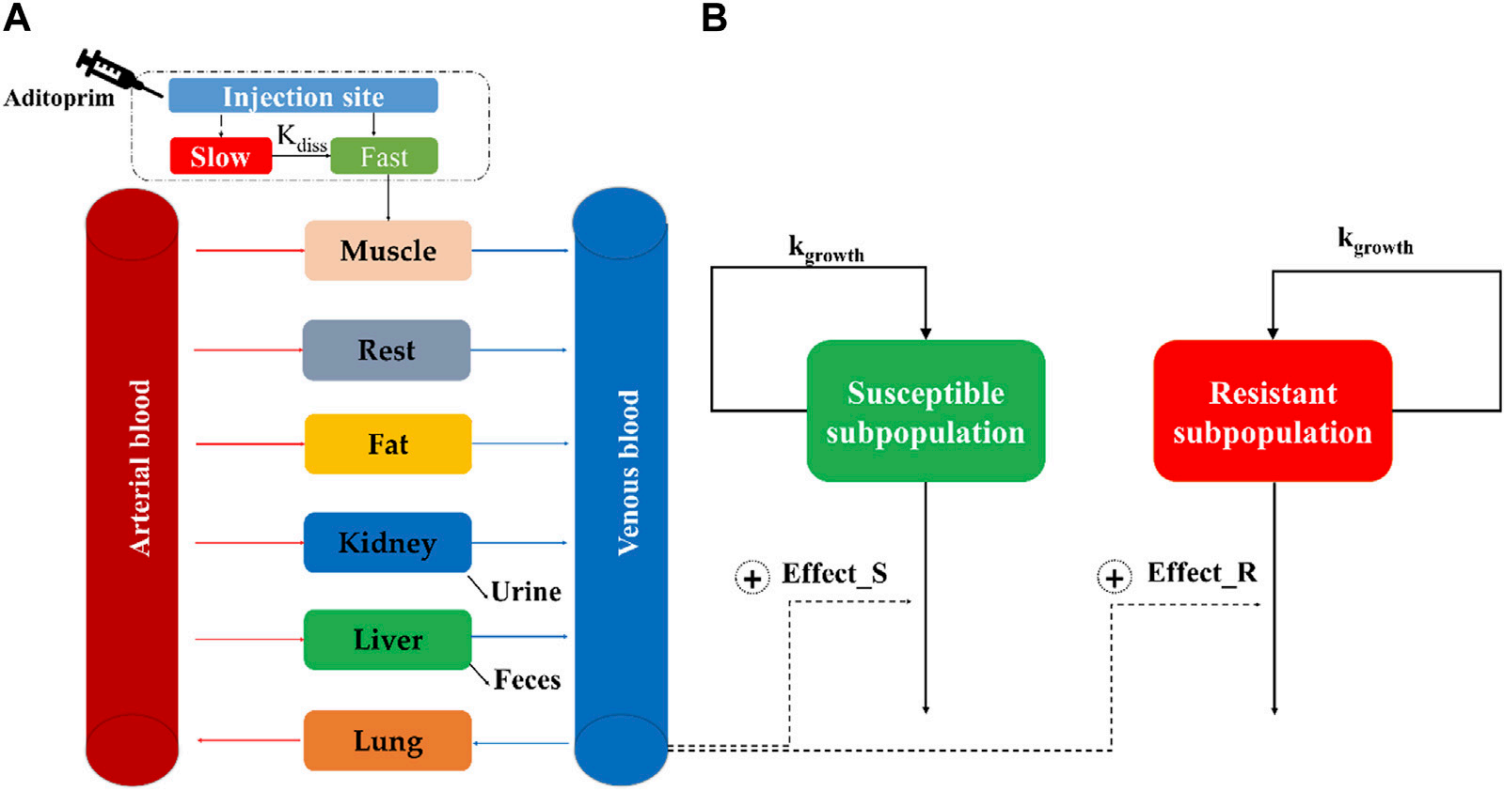
Schematic diagram of the physiologically based pharmacokinetic/pharmacodynamic model for aditoprim against *S. suis* in swines. The structure of the PBPK model and semi-mechanistic PD model is, respectively, described in **(A,B)**.

A seven-compartment PBPK model was structured and connected by the blood circulation system (venous blood and arterial blood) (Figure 1A). For food safety assessment, the major edible tissues, including the liver, kidney, muscle, and fat, were modeled as individual compartments. To simplify the model structure, the rest of the body was considered a pooled compartment.

A two-compartment model is used to describe the drug absorption into muscle after intramuscular injection (Lin et al., 2017). It was divided into slow absorption and fast absorption at the injection site. As shown in Figure 1A, the injection bolus was assumed to have two steps, namely, fast and slow absorption. Fast absorption was assumed to be homogeneously mixed at the injection site and instantaneously available for absorption into the venous compartment with a first-order process (k_im_) (Riad et al., 2021). Slow absorption was described as releasing ADP from the injection site at a first-order rate (*k*_*diss*_) and entering the venous compartment as a fast phase. The excreted tissue is the kidney, and the metabolized tissue is the liver. N-Monodesmethyl-ADP and N-didesmethyl-ADP were found in the tissue; ADP has the highest concentration and the longest duration in tissues, which is regarded as the marker residue. The PBPK model did not describe the metabolism process. *Berkeley Madonna* (version 8.3.23.0; University of California at Berkeley, CA, United States) was used to develop the PBPK model and run all simulations.

### 2.3 Model parameterization and calibration

The PBPK model includes two types of PBPK parameters, namely, physiological and chemical-specific parameters. As for chemical-specific parameters, partition coefficients (PCs) in edible tissues were calculated using the AUC method, as AUC_tissue_/AUC_plasma_, based on the previous study (Wang et al., 2016). These initial PCs were optimized by the residual data. After the model calibration of ADP in tissues, the initial PCs would adjust to more suitable values.

The excreted rate in the kidney and the metabolized rate in the liver were assumed as a first-order constant rate. The urine elimination and liver metabolism rates were assumed as KurineC and KML, respectively. For intramuscular administration, ADP was absorbed into the blood at a first-order constant rate (Kim) under injection dose (doseim). Drugs were distributed into fast absorption (Frac*doseim) and slow absorption [(1-Frac) *doseim]. In slow absorption, the drug transferred to the state of fast absorption at a constant rate (K_diss_) (Riad et al., 2021). All the initial chemical-specific parameter values were calibrated by the curve fitting module in *Berkeley Madonna* and further optimized (Xu et al., 2020).

### 2.4 Validation and sensitivity analysis

The validation dataset is listed in Table 1. All parameters were not changed from those determined by model calibration. Based on the World Health Organization guidelines (WHO, 2010), if the predictive results matched the kinetic profile of the experimental data and were generally within a two-fold difference of the experimental data, then the model could be successfully established. The goodness of fit between observation and simulation was analyzed by linear regression. The determination coefficient (*R* ^2^) values were derived from regression analysis, and the model simulation was acceptable if the value of *R* ^2^ was equal to or higher than 0.75 (Lin et al., 2017). The performance of the PBPK model was assessed by the mean absolute percentage error (MAPE) value, which was less than 50% and considered an acceptable prediction (Yang et al., 2022).

Sensitivity analysis was performed to determine which parameters were important in the selected key model outputs. Each parameter was increased by 10%, and the corresponding AUC_24h_ values of ADP in the liver, kidney, plasma, muscle, and fat were computed. A normalized sensitivity coefficient (NSC) was used to evaluate sensitive parameters, as described in Eq. 1 (Mirfazaelian et al., 2006; Lin et al., 2011).

$$\text{NSC}=\frac{△r}{r}\times \frac{p}{△p},$$ (1)

where *r* is the response variable (AUCs of ADP in plasma and tissues), *Δr* is the change in the response variable, *p* is the original value of the model parameter, and *Δp* is the 10% change in the parameter value. A parameter was considered sensitive when at least one of the absolute values of the NSCs was greater than or equal to 0.25 (Leavens et al., 2014).

### 2.5 Monte Carlo analysis and the populational PBPK model

Considering the population variability of model parameters, a populational (pop) PBPK model was established by integrating the PBPK model and Monte Carlo analysis. Only sensitive parameters were subject to Monte Carlo analysis. Log-normal distributions of model parameters were assumed for chemical-specific parameters, such as tissue partition coefficients. Normal distributions were assumed for physiological parameters, such as bodyweight. The distributions within the lower bound (2.5%) and upper bound (97.5%) were described by the model code, as previously introduced (Li et al., 2018). The coefficients of variance (CVs) for chemical-specific parameters and physiological parameters were defined as default values, 30% and 40%, respectively. Monte Carlo analysis was conducted for *1,000* iterations by bath runs in *Berkeley Madonna*.

### 2.6 Semi-mechanistic PD model

The minimum inhibitory concentration of ADP was determined against ATCC 49619, followed by CLSI documents. Time-killing curves of ADP against ATCC 49619 were conducted in triplicate, as previously described (Mi et al., 2022). These datasets were applied to build a semi-mechanistic PD model. Because of bacterial regrowth under ADP exposure, a two-compartment semi-mechanistic PD model was developed, as shown in Figure 1B (Jacobs et al., 2016). This model includes susceptible subpopulation (Eq. 2) and resistant subpopulation (Eq. 3), which are assumed to be regulated by the natural growth rate and kill rate of an antimicrobial drug. The antibacterial effect was modeled by the E_max_ equation.

$$\frac{dS}{dt}=k_{\text{growth}}\times \frac{\left(S+R\right)}{B_{\max}}\times S-\frac{E_{\max}\times C_{stastic}^{\gamma }}{{EC}_{50_{S}}^{\gamma }+C_{stastic}^{\gamma }}\times S,$$ (2)

where S (CFU/mL) is the bacterial concentration in the susceptible subpopulation; k_growth_, a first-order constant, is described as the bacterial net growth rate. B_max_ is the maximum amount of bacteria in the system; E_max_ (1/h) is the maximum bacteria killed by ADP representing drug efficacy, EC_50_S_ (mg/L) is the concentration of ADP that produces half of the maximum effect of a susceptible subpopulation, γ is a sigmoid coefficient expressing the slope of antimicrobial effect curves and presenting drug sensitivity, and C_stastic_ is the concentration of ADP at time (t).

$$\frac{dR}{dt}=k_{\text{growth}}\times \frac{\left(S+R\right)}{B_{\max}}\times R-\frac{E_{\max}\times C_{stastic}^{\gamma }}{{EC}_{50_{R}}^{\gamma }+C_{stastic}^{\gamma }}\times R,$$ (3)

where R (CFU/mL) is the bacterial concentration in the resistant subpopulation. EC_50_R_ is assumed as the antibacterial effect on the resistant subpopulation.

The estimations of semi-mechanistic PD parameters were operated by the non-linear mixed-effect analysis (*Monolix*, version 2018R1, Lixofit, France). Diagnostic plots, such as goodness fit of prediction versus observation, residuals of IPRED (individual prediction) versus the dependent variables, and time, were adopted to determine whether the model is adequate. In addition, visual predictive check (VPC) was used to validate the prediction of the model.

### 2.7 Application of the PBPK/PD model

Integrated with the PBPK model and semi-mechanistic PD model to build a PBPK/PD model in *Mlxplore* (Lixoft version 2018a, France), the predicted dynamic concentrations from the PBPK model replace the static ADP concentration (C_stastic_) in the semi-mechanistic PD model. Thereby, time courses of the bacterial count and drug concentration under a variety of drug exposures can be simulated. The efficacy of dosage and the best PK/PD parameters related to bacterial response were assessed by the PBPK/PD model. For food safety, a withdrawal interval is determined by the pop-PBPK model.

#### 2.7.1 Determining the best PK/PD parameter

PK/PD parameters, which enable a description of the whole-time course of bacterial kill and growth, can be determined in an *in silico* model (Nielsen and Friberg, 2013). Dose fractions of 0, 2.5, 5, 10, 15, 20, and 25 mg/kg for single administration and twice-daily administration were inputted in the PBPK/PD model. Concentration–time curves of ADP and bacterial count–time curves can be simulated. PK/PD parameters (AUC/MIC and %T > MIC) are directly calculated by the PBPK model (Mi et al., 2023). The cumulative area under the curve of the total bacterial count over 24 h (AUC_0-24h (Bacterial count)_) was used as a bacteriological effect (Pelligand et al., 2019; Ronaghinia et al., 2021). PK/PD parameters (AUC/MIC and %T > MIC) versus the bacteriological effect, AUC_0-24h (Bacterial count)_, were fitted with an inhibitory sigmoid model (Eq. 4). Curve fitting was performed in *Phoenix* (version 8.3, Certara, United States), and the best PK/PD parameter related to bacteriological effect was selected.

$$E=E_{0}-\frac{I_{\max}\cdot {INDEX}^{N}}{{INDEX}^{N}+{{INDEX}_{50}}^{N}},$$ (4)

where *E*_*0*_ was the effect under a drug concentration of zero. The maximum possible observed effect is I_max_. INDEX was the value of the PK/PD parameters (AUC_24h_/MIC or %T > MIC). INDEX_50_ was the value of AUC_24h_/MIC or %T > MIC, producing a 50% reduction in I_max_, and N was the Hill coefficient that described the steepness of the curve.

#### 2.7.2 Dosage optimization

By the PBPK/PD model, the time courses of bacterial count can be, respectively, simulated under the dosages of 5, 12.5, 15, and 20 mg/kg twice a day. The bacterial count change curves of the susceptible subpopulation, the resistant subpopulation, and the total are presented. Based on the antibacterial effect, the dosage can be determined.

#### 2.7.3 Withdrawal interval assessment

For food safety assessment, the withdrawal interval needs to be determined. The datasets of 1,000 iterations are simulated by the pop-PBPK model. The concentration–time curves of the 1st percentile, median, and 99th percentile in the liver and kidney were presented. The MRL of ADP in the liver is 0.303 ug/g and in the kidney is 0.084 ug/g (Wang et al., 2016). The 99th percentile curve is used to compare with the maximum residue limits to determine the withdrawal intervals. The time point when the ADP concentration falls under the MRL is selected to determine the withdrawal interval (Li et al., 2017).

## 3 Result

### 3.1 PBPK model calibration

**FIGURE 2:**
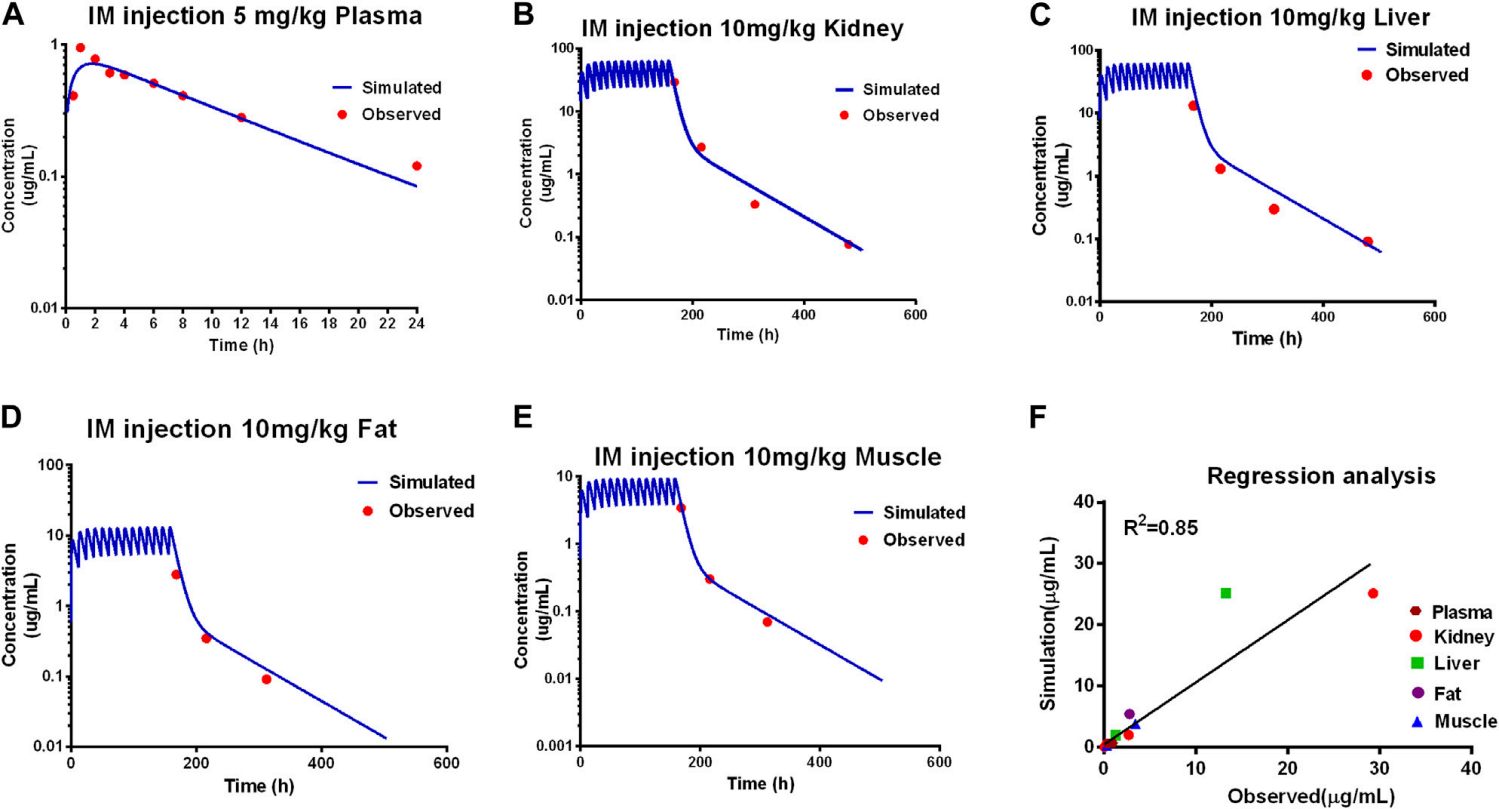
Calibration of the PBPK model. Comparison of model prediction (blue line) and observed data (red dot) in plasma **(A)** via 5 mg/kg, and in the kidney **(B)**, liver **(C)**, fat **(D)**, and muscle **(E)** via IM repeated 14 doses at 10 mg/kg. The *R* ^2^ value was 0.86 of the regression analysis **(F)**.

Model predictions of ADP concentrations in plasma and edible tissues at different time points after the administration were compared with the observed concentrations for different dosage regimens, as shown in Figure 2.

### 3.2 PBPK model validation and evaluation

**FIGURE 3:**
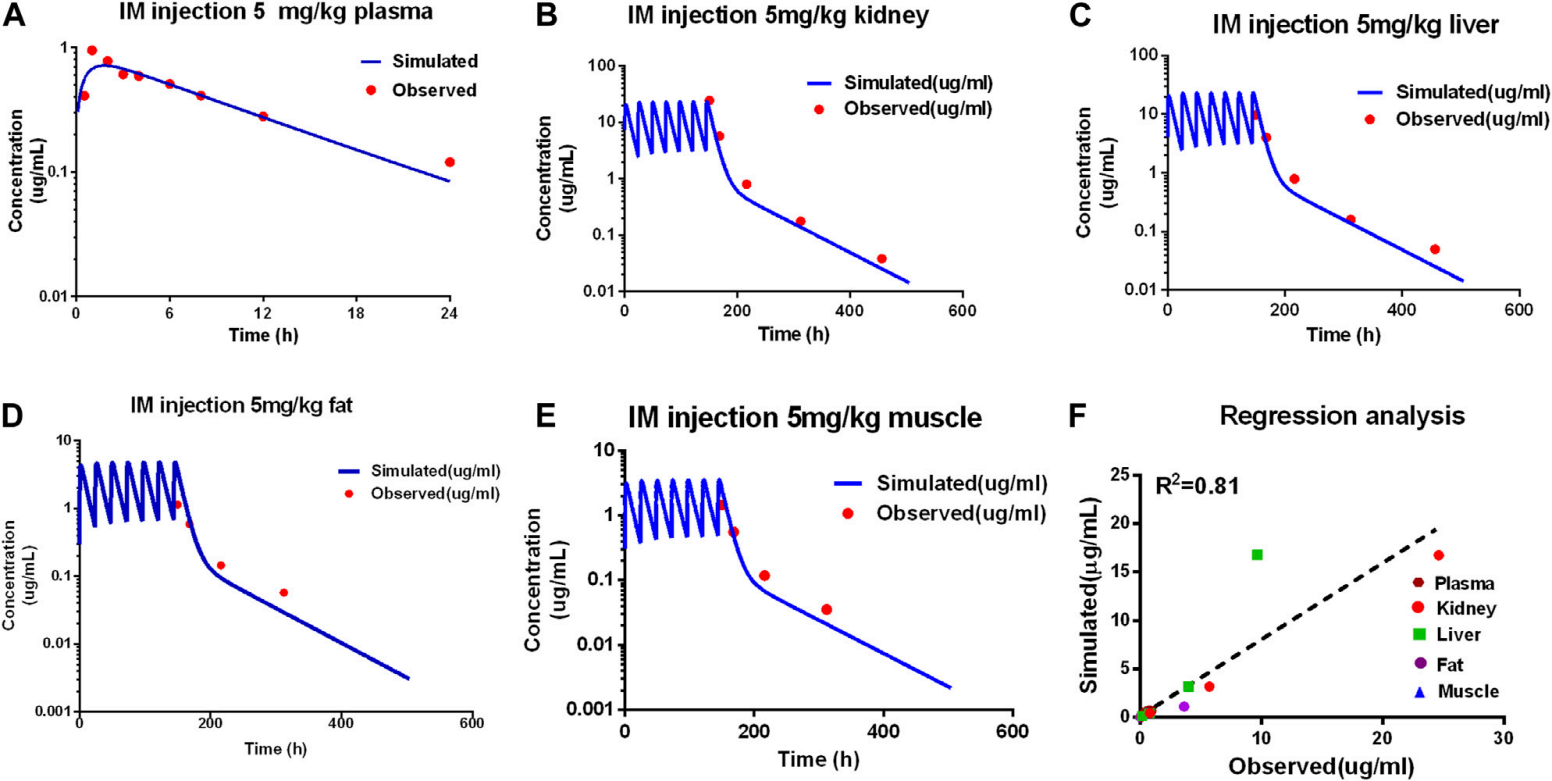
Evaluation of the PBPK model. Comparison of model prediction (blue line) and observed data (red dot) in plasma **(A)** via 5 mg/kg, and in the kidney **(B)**, liver **(C)**, fat **(D)**, and muscle **(E)** via IM repeated 7 doses at 5 mg/kg. The *R* ^2^ value was 0.81 of the regression analysis **(F)**.

Comparing the observed concentration of ADP in the plasma, liver, kidney, muscle, and fat with the model prediction from the validation dataset, good consistency between the observed data with model prediction is shown in Figure 3. The goodness of fit was evaluated using the determination coefficients (*R* ^2^). The value of *R* ^2^ between measured and simulated concentrations of ADP in edible tissues and plasma was 0.81 for the PBPK model. Except for the predicted concentration at the first time point of fat (6 h after administration) being out of the two-fold difference of observed data, all are within the two-fold difference. For the determination of withdrawal intervals, at the later time points, simulated versus measured concentrations for ADP need to be similar. The liver and kidney are assumed as residual target tissues, and the elimination of ADP in the liver or kidney is slower than that in other tissues (Wang et al., 2016). If the model can accurately predict the ADP concentration in the liver and kidney, it can be used in the prediction of withdrawal intervals. The results of MAPE show that the prediction of ADP in plasma and edible tissues, in addition to fat, is acceptable (Supplementary Figure S1).

Overall, the PBPK model can accurately describe the kinetic of ADP concentrations in tissues and plasma. The PBPK parameters are shown in Table 2.

**TABLE 2:** Physiological parameters of swines and chemical-specific parameters of ADP used in the PBPK model.

Parameter (unit)	Abbreviation	Mean	Resource
Bodyweight (kg)	BW	30	—
Cardiac output (L/h/kg)	QCC	4.944	Upton (2008)
Organ blood flow (% of QCC)			
Muscle	QMC	0.2524	Upton (2008)
Rest	QRC	0.3055	Calculated
Liver	QLC	0.3053	Upton (2008)
Kidney	QKC	0.1398	Upton (2008)
Lung	QLUC	1	Upton (2008)
Organ volume (% of BW)			
Lung	VLUC	0.01	Upton (2008)
Muscle	VMC	0.4	Upton (2008)
Rest	VRC	0.232	Calculated
Liver	VLC	0.0294	Upton (2008)
Kidney	VKC	0.004	Upton (2008)
Arterial blood	VartC	0.016	Upton (2008)
Venous blood	VvenC	0.044	Upton (2008)
Tissue to blood partition coefficient			
Liver	PL	5.249	Calculated/optimized
Kidney	PK	6	Calculated/optimized
Muscle	PM	0.79	Calculated/optimized
Fat	PF	1.1	Calculated/optimized
Rest	PT	0.18	Calculated/optimized
Absorption rate constant (/h)			
	Kim	1.3	Model fitting
	Frac	0.92	Model fitting
	Kdiss	0.0118	Model fitting
Hepatic clearance (L/h/kg)	KML	0.01	Model fitting
Renal clearance (L/h/kg)	KurineC	0.1	Model fitting
Percentage of plasma protein binding	PB	0.82	Model fitting

### 3.3 Sensitivity analysis and Monte Carlo simulation

The AUCs were insensitive to all physiological parameters. The AUCs of ADP in four edible tissues were positively related to partition coefficients with NSC values of 1. The renal clearance rate (KurineC) and the fraction of the dose allocated to fast absorption (Frac) influence the AUC_24h_ in all edible tissues and plasma. The protein-binding rate only influences the AUC in plasma. All the sensitive parameters described above were included in the Monte Carlo analysis. The values and distributions of parameters used in Monte Carlo analysis are provided in Table 3.

**TABLE 3:** Values and distributions of parameters used in Monte Carlo analysis for the PBPK model.

Parameter	Distribution	Mean	CV	SD	Lower	Upper
PL	Lognormal	5.249	0.40	2.0996	2.29	10.37
PK	Lognormal	6.00	0.40	2.4	2.62	11.85
PM	Lognormal	0.79	0.40	0.316	0.34	1.56
PF	Lognormal	1.10	0.40	0.44	0.48	2.17
KurineC	Lognormal	0.10	0.40	0.04	0.04	0.20
Frac	Lognormal	0.92	0.10	0.092	0.75	1.00
PB	Lognormal	0.82	0.40	0.328	0.36	0.99

PL,PK,PM and PF respectively represent liver, kidney, muscle and fat to blood partition; Frac represents fraction of intramuscular doses allocated to fast absorption; PB represents protein binding rate; CV represents coefficients of variance; SD represents standard deviation; Lower represents lower bounds of statistical distribution; Upper represents upper bounds of statistical distribution.

### 3.4 Semi-mechanistic PD model

**FIGURE 4:**
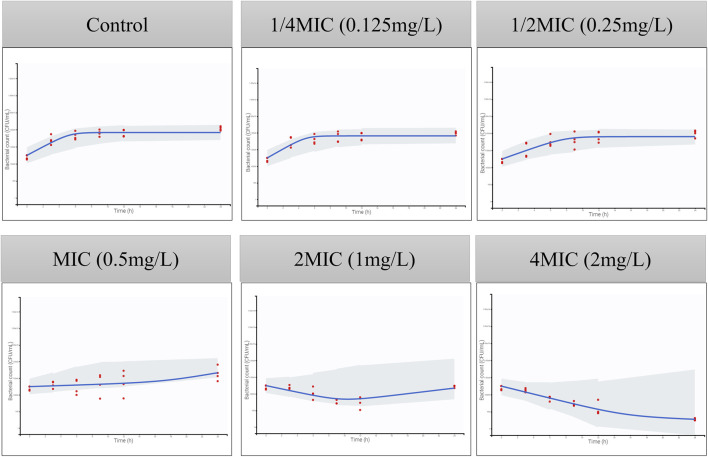
Visual predictive checks for the bacterial count in time-killing curves. The red circles correspond to the observed data, while the blue lines correspond to the median simulated data. The shaded area corresponds to the 95% confidence interval (CI) around the simulated median data. The y-axis represents the Log_10_ bacterial count (CFU/mL), and the x-axis represents time (h).

The MIC of ADP against ATCC 49619 is 0.5 ug/mL, followed by CLSI documents (CLSI, 2018). The parameters of the semi-mechanistic PD model are shown in Table 4. The maximum effect of ADP was 1.45 h^−1^, which was 1.74 folds of the net bacterial growth rate of 0.833 h^−1^. To achieve half of the maximum effect for the susceptible subpopulation (EC_50_S_), the ADP concentration needs to reach 0.685 ug/mL. In addition, to achieve half of the maximum effect for the resistant subpopulation (EC_50_R_), the ADP concentration needs to reach 1.63 ug/mL, which was about 2.5-fold to EC_50_S_. Model fits for bacterial time courses are shown in Figure 4. For the model evaluation, a core set of diagnostic plots is shown in Supplementary Material.

**TABLE 4:** Parameters for the semi-mechanistic PD from the time-killing curves of ADP against *S. suis*.

Parameter	Unit	Explanation	Value (R.S.E. %)
K_growth_	1/h	Rate constant of net natural bacterial growth	0.833 (15.6)
MF	—	Mutation frequency	10^–3^ (fixed)
B_max_	CFU/mL	Bacterial count in the stationary phase	4.61*10^7^ (−)
E_max_	1/h	Maximum kill rate constant	1.45 (4.47)
EC_50_S_	mg/L	Antibacterial concentration that produces 50% of E_max_ for the susceptible population	0.685 (8.12)
EC_50_R_	mg/L	Antibacterial concentration that produces 50% of E_max_ for the resistant population	1.63 (4.2)
γ	—	Hill factor	2.5 (6.5)

### 3.5 Model application

#### 3.5.1 PK/PD parameters

**FIGURE 5:**
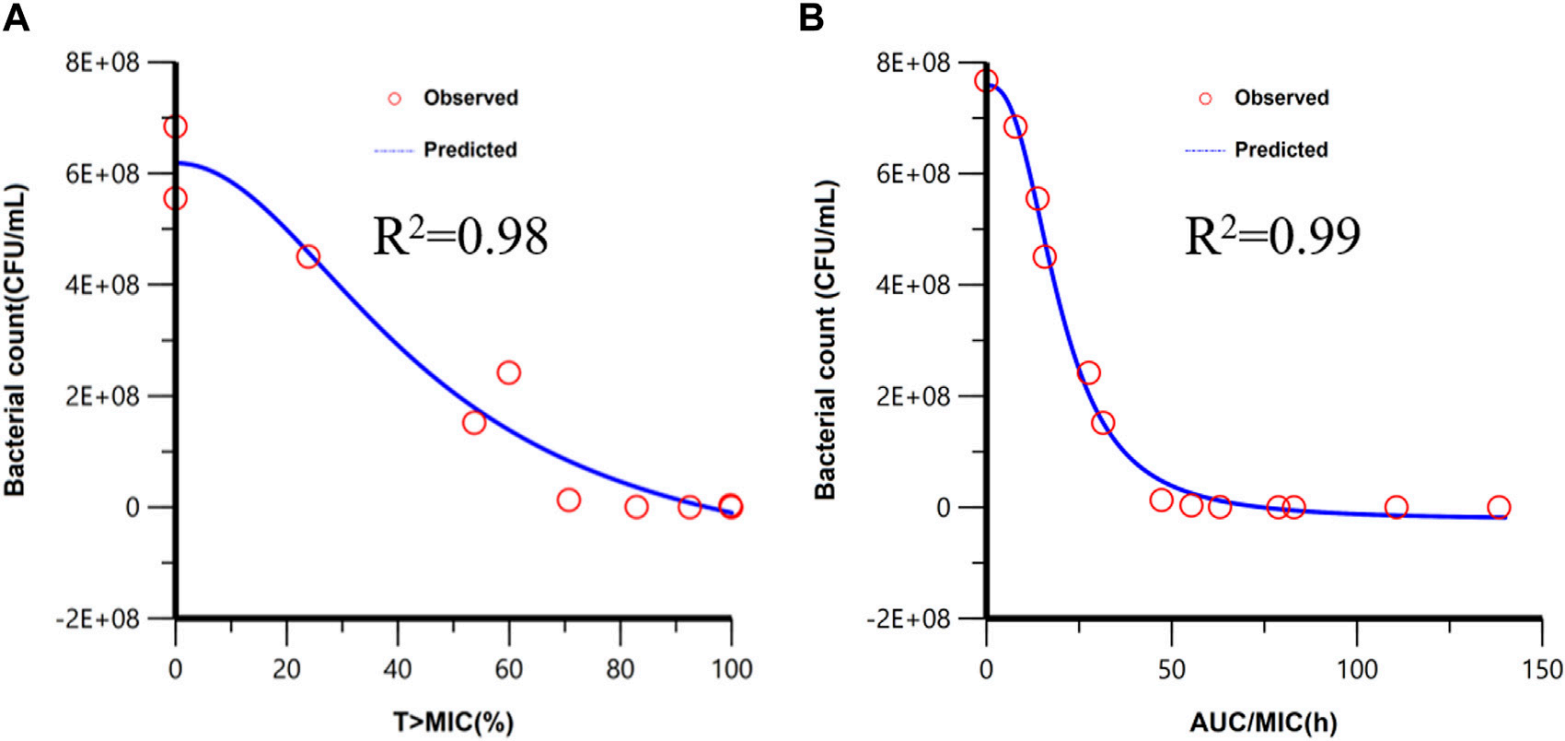
Fitting of the I_max_ sigmoid model for the bacteriological effect [AUC_0-24h (bacterial count)_] with T > MIC **(A)** and AUC/MIC **(B)**. Red circles are the AUC_0-24h (bacterial count)_ values under different dose exposure.

The PBPK/PD model is applied to simulate the time courses of the bacterial count under different dosages, which are shown in Supplementary Result. T > MIC and AUC/MIC of different dosage regimens were calculated by the PBPK model. The PK/PD relationship between PK/PD parameters and AUC_0-24h (Bacterial count)_ was conducted in *Phoenix*, and the PK/PD relationships are shown in Figure 5. The goodness-of-fit values were better for *f*AUC/MIC (*r* ^2^ = 0.99) than for *f* T > MIC (*r* ^2^ = 0.98). Similar to the result of the empirical PK/PD model (Qu et al., 2022), AUC/MIC is also the best PK/PD parameter.

#### 3.5.2 Assessment of the dosage regimen

**FIGURE 6:**
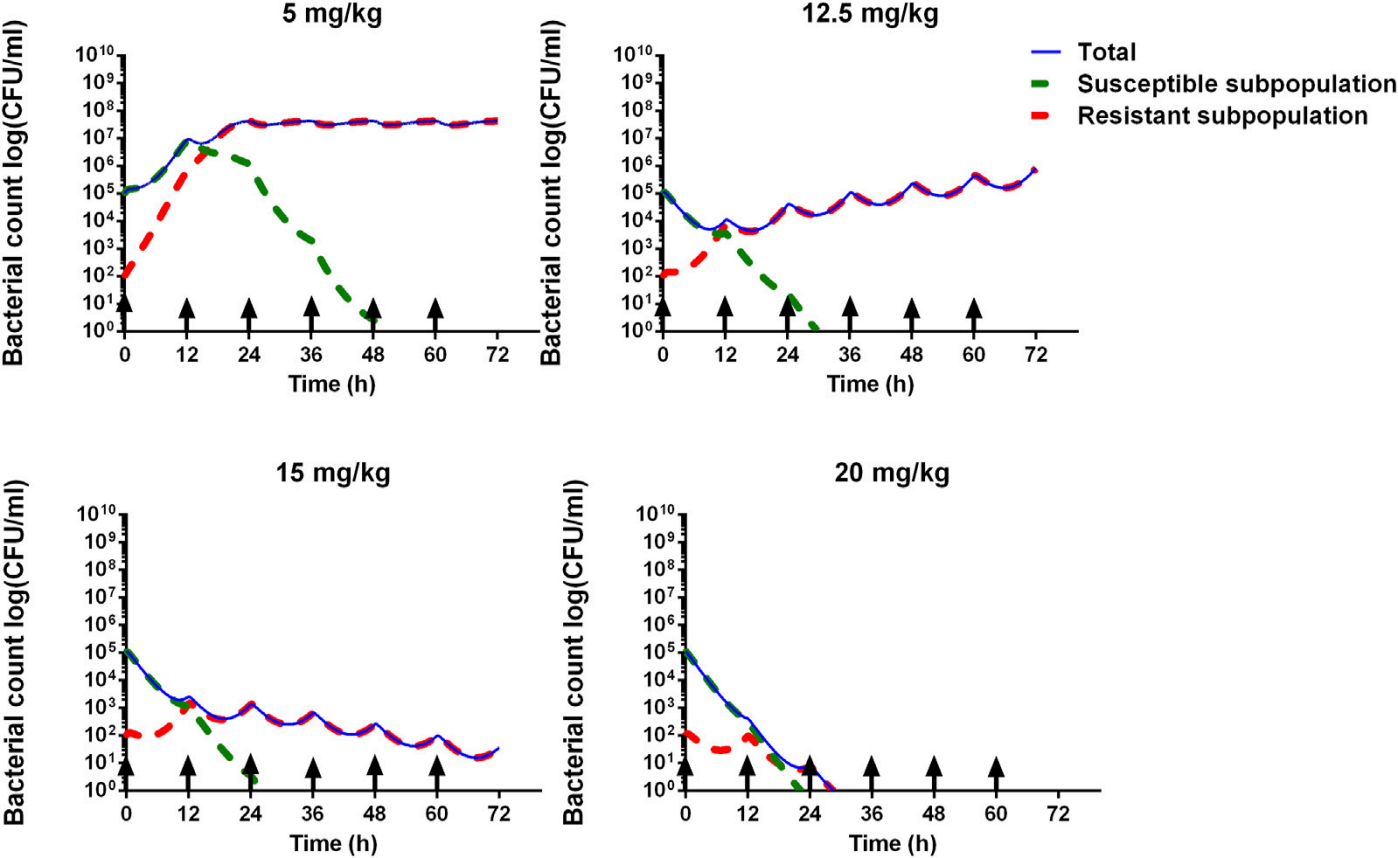
Model prediction of *S. suis* for different ADP doses (5, 12.5, 15, and 20 mg/kg) with 12 administrated intervals for 3 consecutive days. The blue line represents the total bacterial count. The green dashed line represents the susceptible subpopulation. The red dashed line represents the resistant subpopulation. The black arrow means administered time.

The time courses of different bacterial subpopulations, total, susceptible subpopulation, and resistant subpopulation were simulated under different dosage regimens. As shown in Figure 6, while the dose was 5 mg/kg, the susceptible subpopulation gradually decreased and the resistant subpopulation increased, after 48-h time points, almost a resistant subpopulation in the total bacterial system. The dose of 12.5 mg/kg can accomplish the bacteriostat action. The dose of 15 mg/kg can reach the bacterial reduction of approximately 3-log_10_ CFU/mL and inhibit the growth of resistant subpopulations. At an ADP dose of 20 mg/kg, the susceptible and resistant subpopulations rapidly declined after being twice administered, and bacteria were eliminated.

#### 3.5.3 Withdrawal intervals

**FIGURE 7:**
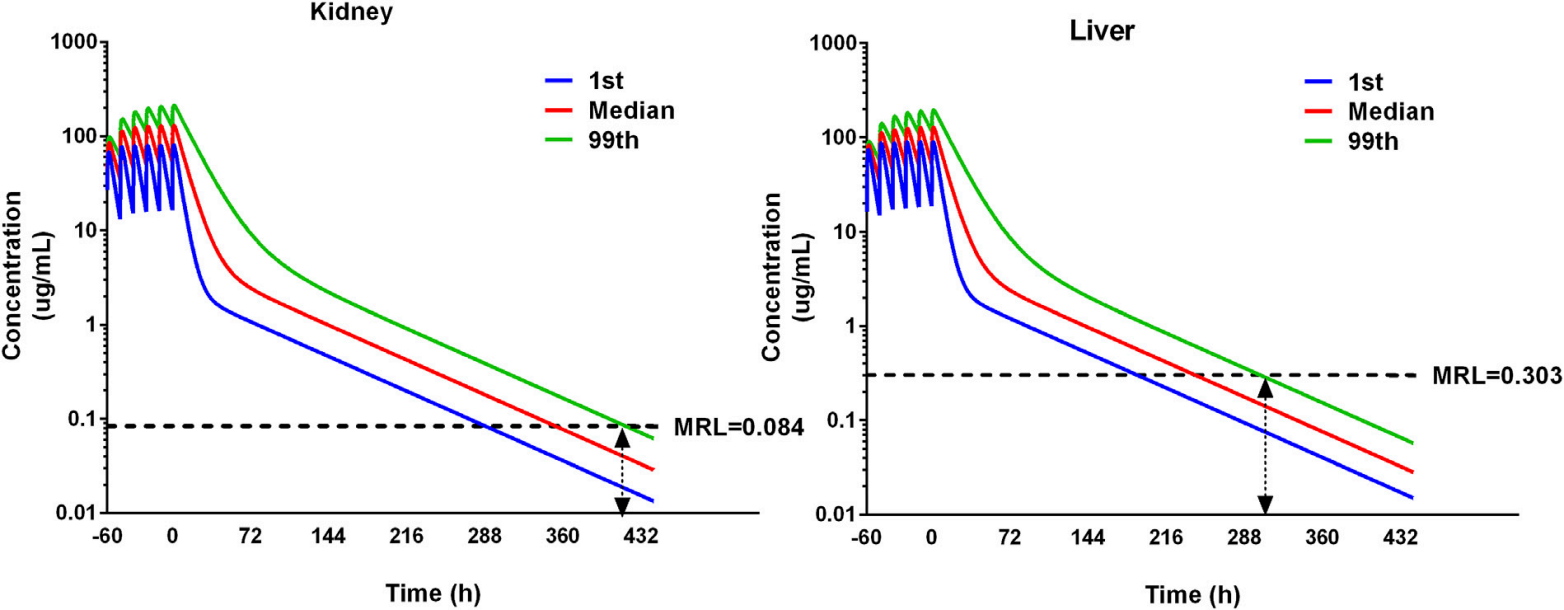
Simulation of ADP concentrations in the kidney and liver using the pop-PBPK model. The dose regimen is 20 mg/kg with 12 intervals for 3 consecutive days. The median and 1st and 99th percentiles of simulated results were plotted. The maximum residual limitation (MRL) is shown on each tissue using black dash lines. The withdrawal intervals were determined when the concentration in different tissues is below MRL for the 99th percentile of the population. The double arrow means the concentration at that time point is below the MRL.

The residual depletion rates in the kidney and liver are the slowest, and they are selected as target residual tissues. Concentration–time curves of 1,000 iterations in the kidney and liver were simulated. The median and 1st and 99th percentiles of 1,000 iterations were selected and are shown in Figure 7. The MRL of ADP in swine has been investigated previously (Wang et al., 2016); in the liver it is 0.303 ug/g, and in the kidney, it is 0.084 ug/g. Compared with MRL, the WDI is conservatively determined to be 12.6 days in the liver and 17.4 days in the kidney. Note that if the estimated WDI was a fraction of a day, it was rounded up to the next whole day. Therefore, the WDI after 20 mg/kg with 12 intervals for 3 consecutive days is 18 days.

## 4 Discussion

*S. suis* is the highest isolated common bacterial pathogen in Chinese pig farms (Zhang et al., 2019). Given its virulence in humans and animals, it poses a significant threat to public health. Our group has successfully developed an empirical PK/PD model to determine PK/PD parameters for various antibacterial effects and dosage regimens of ADP injection against *S. suis*. However, the empirical PK/PD model exhibited several limitations, as mentioned above. In this manuscript, we have further adopted a PBPK/PD model to determine an optimized dosage of ADP and a withdrawal interval.

The PBPK model is a mechanism-based simulation process that describes the absorption, distribution, metabolism, and elimination of drugs in the body by considering the relationship among physiological, biochemistry, anatomical, and compound properties. In recent years, PBPK models have been widely used in various fields, including drug discovery and development (Rowland et al., 2011; Jones et al., 2015), risk assessment of environmental chemicals (Lu et al., 2016; Tan et al., 2018), safety assessment of animal-derived food products (Lin et al., 2016; Henri et al., 2017), and residue prediction of veterinary drugs in various edible tissues (Viel et al., 2023). The advantages of PBPK models in the prediction of drug residues in edible tissues in a diverse population of food animals make them an ideal tool in veterinary drug residue monitoring. In the current study, the PBPK model is calibrated and validated with two independent datasets. Based on the result of model evaluation including the visual comparison between the observed and predicted data, *R* ^2^, and RMSE, the prediction of the PBPK model is acceptable and reliable. By inserting Monte Carlo analysis into the PBPK model, a pop-PBPK model can be developed and applied to predict the drug concentration in 1,000 virtual animals, and the withdrawal interval can be determined.

The semi-mechanistic PD model was established by mathematical equations to capture the time-killing curves and elucidate bacterial growth, bacterial death, and antibacterial effects under antibiotic exposure. It can integrate with the PBPK model to develop the PBPK/PD model. A PBPK/PD model of ceftiofur against *Pasteurella multocida* was established to validate the dosage regimen (Mi et al., 2022). A whole-body PBPK/PD model of ciprofloxacin was developed to predict the antibacterial effect in different tissues (Sadiq et al., 2017). In addition, it was also applied to the definition of the PK/PD breakpoint (Iqbal et al., 2020). Upon examining the time-killing curves of ADP against *S. suis*, bacterial regrowth was observed (Figure 4). A model consisting of susceptible and resistant subpopulations was adopted. The value of EC_50_ is used to describe the sensitivity of different subpopulations to ADP. The EC_50_S_ is smaller than EC_50_R_, which means it is more easily killed by antimicrobial agents. Based on the goodness of fit, residual plots, and VPC, the semi-mechanistic PD model can describe the time courses of bacterial growth under ADP exposure.

The PBPK/PD model was integrated with the PBPK model and semi-mechanistic PD model, and it can simulate bacterial growth under ADP exposure. ADP was determined at a dose of 4.1 mg/kg against *S. suis* by the empirical PK/PD model. However, in our paper, by the PBPK/PD model, *S. suis* cannot be eradicated, and the resistant subpopulation is still alive under a dose of 5 mg/kg/12 h (Figure 6). The dose of 20 mg/kg is much better because it can perform antibacterial effects and inhibit resistance. ADP has turned out to be less toxic and not mutagenic (Wang et al., 2015a; Wang et al., 2015b). The preclinical study has stated that ADP can perform an antibacterial effect on swine *Streptococcus* (Cheng et al., 2017). Given its status as a novel veterinary drug, further clinical development, specifically in phases II and III, is imperative.

In this study, we adopt a model-informed drug development (MIDD) approach to determine the dose that was employed in the clinical trial. In addition, it is expected to apply the hollow fiber infectious model to monitor the resistance change under drug exposure, which can support clinical usage.

## Data Availability

The original contributions presented in the study are included in the article/Supplementary Material; further inquiries can be directed to the corresponding author.
