# Supplementary material for: A physiologically based pharmacokinetic/pharmacodynamic model to determine dosage regimens and withdrawal intervals of aditoprim against Streptococcus suis
Source: Front Pharmacol. 2024 Apr 17;15:1378034. doi: 10.3389/fphar.2024.1378034 (PMC11061430; doi:10.3389/fphar.2024.1378034)
Supplement: Supplementary file 1 [file DataSheet1.pdf]

Supplementary Material

1 Supplementary Figures

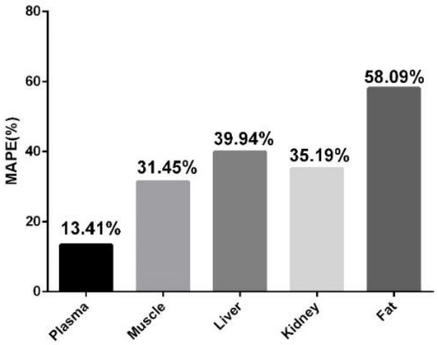

Supplementary Figure 1. The mean absolute percentage error (MAPE) analysis for results of the model evaluation. The MAPE values for the plasma, muscle, kidney, liver and fat and were shown. Besides Fat compartment, the MAPE for other compartment is low of 50%.

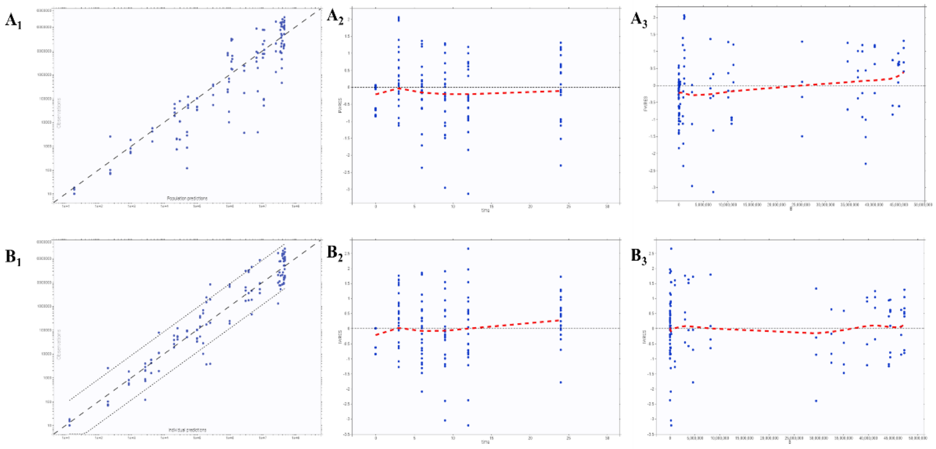

Supplementary Figure 2 Core set of diagnostic graphs for semi-mechanistic PD model. A1: Plot of Observation vs. population predictions. A2 :Plot of population weighted residual vs. time. A3 :Plot of population weighted residual vs. prediction. B1: Plot of Observation vs. individual predictions. B2: Plot of individual weighted residual vs. time. B3: Plot of individual weighted residual vs. prediction; Blue dots represent observed data.

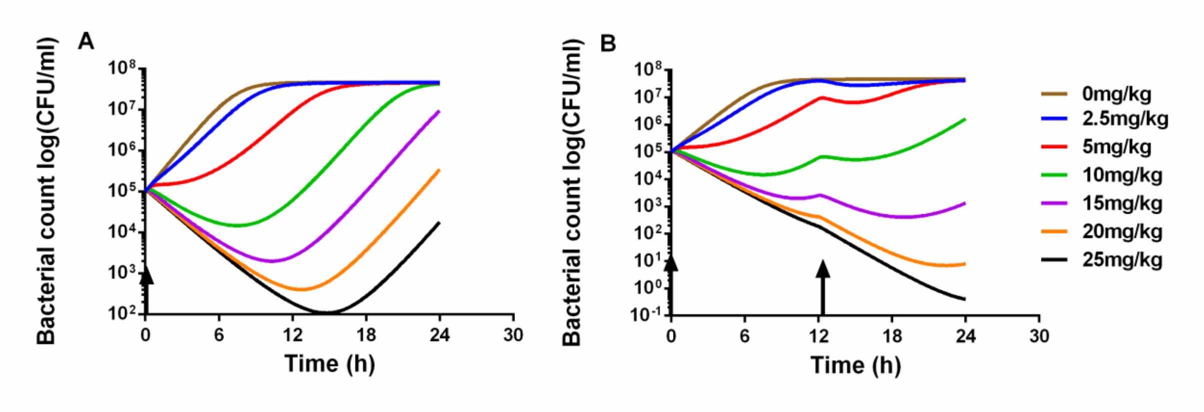

Supplementary Figure 3 Escalating dose plots of ADP dosage regimens with different administrated intervals. A represents the one administration for various doses during the 24 hours; B represents the two administrations for various doses during the 24 hours. The black arrow means administrated time.

2 Supplementary Tables

Supplementary Table 1 Concentrations of ADP in swine edible tissues (μg/kg) after intramuscular injection at 10 mg/kg twice daily for 7 consecutive days(Wang, 2020).

Tissue	Time(day)				
	0.5	3	7	14	28
**Liver**	13311.0±1691.9	1305.4±151.1	255.7±42.4	90.4±7.2	ND
**Kidney**	29283.5±4005.9	2709.6±661.5	308.5±23.4	77.7±7.4	ND
**Muscle**	3439.1±434.8	302.6±34.9	69.7±9.2	ND	ND
**Fat**	2524.7±262.8	347.9±46.4	82.4±8.5	ND	ND

Note: ND represents not detectable.

Supplementary Table 2 Concentrations of ADP in swine edible tissues (μg/kg) after intramuscular injection at 5 mg/kg once daily for 7 consecutive days (Wang, 2016).

Tissue	Time(day)				
	0.25	1	3	7	14
**Liver**	9691.6±1437.8	4014.6±177.4	989.5±200.1	160.3±54.3	69.1±19.0
**Kidney**	28785.6±4140.3	6504.9±777.0	1014.6±214.1	177.8±51.6	38.6±14.1
**Muscle**	1751.6±197.7	555.2±93.1	118.0±31.0	44.5±9.2	ND
**Fat**	1027.9±114.4	586.8±59.8	144.8±30.3	65.5±8.2	ND

Note: ND represents not detectable.

Supplementary Table 3 The mathematical equation of PBPK model

Tissue	Mathematical equation
Venous blood	$\mathrm{V}_{vb} * \frac{\mathrm{dC}_{vp}}{dt} = Q_{muscle} * \left(\frac{\mathrm{C}_{\mathrm{muscle}}}{PM}\right) + Q_{rest} * \left(\frac{\mathrm{C}_{\mathrm{rest}}}{\mathrm{PR}}\right) + Q_{liver} * \left(\frac{\mathrm{C}_{\mathrm{liver}}}{\mathrm{PL}}\right) + Q_{kidney} * \left(\frac{\mathrm{C}_{\mathrm{kidney}}}{\mathrm{PK}}\right) + Q_{fat} * （\frac{C_{fat}}{PR}） - Q_{Cc} * C_{vb}$
Arterial blood	$\mathrm{V}_{ab} * \frac{\mathrm{dC}_{ap}}{dt} = Q_C \times (C_{vb} - \mathrm{C}_{ap})$
Muscle	$\mathrm{V}_{muscle} * \frac{\mathrm{dC}_{muscle}}{dt} = Q_{muscle} * （C_{ap} - \frac{C_{muscle}}{PM}）$
Fat	$\mathrm{V}_{fat} * \frac{\mathrm{dC}_{fat}}{dt} = Q_{fat} * （C_{ap} - \frac{C_{fat}}{PF}）$
Kidney	$\mathrm{V}_{kidney} * \frac{\mathrm{dC}_{kidney}}{dt} = Q_{kidney} * \left(C_{ap} - \frac{C_{kidney}}{\mathrm{PK}}\right) - KurineC \times BW \times \frac{C_{kidney}}{\mathrm{PK}}$
Liver	$\mathrm{V}_{liver} * \frac{\mathrm{dC}_{liver}}{dt} = Q_{liver} * \left(C_{ap} - \frac{C_{liver}}{PL}\right) - \mathrm{KML} \times BW \times \frac{C_{liver}}{\mathrm{PL}}$
Rest	$\mathrm{V}_{rest} * \frac{\mathrm{dC}_{rest}}{dt} = Q_{rest} * （C_{ap} - \frac{C_{rest}}{PR}）$

Note: C_x (μg/mL) is the concentration of ADP in each compartment; V_x and Q_x are the volume (kg) and blood flow (L/h) through a tissue, respectively; Subscript x means the compartment name. Q_{cc} (L/h) represent the cardiac output; BW represents bodyweight; PM, PF,PK,PL,PR represent the tissue partition coefficient of muscle, fat, kidney, liver and rest, respectively; KurineC (L/h/kg) mean renal clearance; KML (L/h/kg) mean hepatic clearance

3 Model Code

3.1 Code of of ADP PBPK model

```
METHOD RK4
STARTTIME = 0
STOPTIME=24
DT = 0.01
DTOUT = 0.1

;Dosing
PDOSEoral=0;
PDOSEiv=0;
PDOSEim=10;

; Dosing, repeated doses
tinterval1 =24; Varied dependent on the exposure paradigm (h)
Tdoses1=14; The number of injections for multiple IM
dosingperiod = if time < Tdoses1*tinterval1-DT then 1 else 0

; Parameters for exposure scenarios
DOSEoral = PDOSEoral * BW; (mg)
DOSEiv=PDOSEiv * BW
DOSEim=PDOSEim * BW

; Physiological parameters
; Blood flow rates

BW=30;
QCAR=4.944;
QLC=0.3053;
QKC=0.1398;
QMC=0.2524;
QFC=0.1747;

; Tissue/Organ volumes

VLC=0.0294;
VKC=0.004;
VMC=0.4;
VFC=0.3;
VBC=0.06;

; Mass Transfer Parameters (Chemical-specific parameters)
; Partition coefficients (PC, tissue:plasma)

PL=4. ;
PK=5;
PM=0.75;
PF=1;
POT=0.26;

; Cardiac output and blood flows to tissues(L/h)
```

```
QC=QCAR*BW
QL=QLC*QC
QK=QKC*QC
QM=QMC*QC
QF=QFC*QC
QOT=QC-(QL+QK+QM+QF)

; Tissue/Organ volumes
VL=VLC*BW
VK=VKC*BW
VM=VMC*BW
VF=VFC*BW
Vblood=VBC*BW
VOT=BW-(VL+VK+VM+VF+Vblood)

; Oral absorption Constant
Ka =0.046;
Kst = 0.25 ;/h, gastric emptying rate constant
Kf=0.005;
KML=0.01;

{Kinetic Constants}
; IM Absorption Rate Constants
Kim = 1.3 ; /h, IM absorption rate constant
Frac = 0.89 ;
Kdiss = 0.0115 ;/h
PB=0.87;

F=0.18;

;Urinary elimination rate constant adjusted by bodyweight
KurineC =0.11
;Urinary elimination rate constant
Kurine = KurineC * BW ;L/h

; Concentration of the chemical in vein compartment
CVL = AL/(VL * PL)
CVK = AK/(VK * PK)
CVF = AF/(VF*PF)
CVOT = AOT/(VOT * POT)
CVM = AM/(VM * PM)


;IM dosing model
; Dosing, IM, intramuscular

Rinputim = pulse(DOSEim,0,tinterval1)*dosingperiod
Rpenim = Rinputim*(Frac);
Rppgim =  Rinputim*(1-Frac);
Rim = Kim*Amtsiteim
d/dt(Absorbim) = Rim
```

```
init Absorbim = 0
d/dt(Amtsiteim) = Rpenim- Rim + Kdiss* DOSEppgim
init Amtsiteim = 0
d/dt(DOSEppgim) = Rppgim-Kdiss* DOSEppgim
init DOSEppgim = 0


 ;con' of chemical in the vein
CV = ((QL*CVL+QK*CVK+QM*CVM+QOT*CVOT+QF*CVF+Rim)/QC)
d/dt(AUCCV) = CV
init AUCCV = 0

RA = QC*(CV-CA)
d/dt(AA) = RA
init AA = 0

CA = AA/Vblood
CAfree=CA*(1-PB)

;DVD in liver
RL=QL*(CA-CVL)-RML
d/dt(AL)=RL
init(AL)=0

RML=KML*CVL*BW
d/dt(AML)=RML
init(AML)=0

CL=AL/VL
d/dt(AUCCL)=CL
init(AUCCL)=0


; Urinary excretion of OTC
Rurine = Kurine * CVK
d/dt(Aurine) = Rurine
init(Aurine)=0

;kidney
RK = QK * (CA - CVK) - Rurine
d/dt(AK) = RK
init(AK)=0

CK = AK/VK ; con' of chem in kieney
d/dt(AUCCK) = CK
init(AUCCK)=0


;DVD in muslce compartment
RM = QM * (CA - CVM)
d/dt(AM) = RM;amount of chemical in muscle
init(AM)=0
```

```
CM =AM/VM
d/dt(AUCCM) = CM
init(AUCCM)=0

;DVD in fat compartment
RF=QF*(CA-CVF)
d/dt(AF) = RF
init(AF)=0

CF=AF/VF
d/dt(AUCCF) = CF
init(AUCCF)=CF

; DVD in SPT of body compartment
ROT=QOT*(CA-CVOT)
d/dt(AOT)=ROT
init(AOT)=0

COT=AOT/VOT
d/dt(AUCCOT) = COT
init(AUCCOT)=0

; Mass balance
Qbal = QC - QL - QK - QM - QF - QOT
Tmass = AA + AL + AK  + AM + AF + AOT+ Aurine+AML
Bal = Absorbim- Tmass
```

3.2 Code of Semimechanistic PD model

```
DESCRIPTION:
 - PK  one compartment model defined using pkmodel
[LONGITUDINAL]
input =  {Cc,Emax,gama,EC50_E,kg,Bmax,EC50_R}
Cc = {use=regressor}
EQUATION:
odeType = stiff
E_0 =99900
R_0=100
ddt_E = kg*(1-(E+R)/Bmax)*E-((Emax*Cc^gama)/(Cc^gama+EC50_E^gama))*E
ddt_R=kg*(1-(E+R)/Bmax)*R-((Emax*Cc^gama)/(Cc^gama+EC50_R^gama))*R

B=E+R
OUTPUT:
output =B
```

4. Reference

Wang, L. (2016). Disposition and residue depletion of aditoprim in pig,chickne,carp and rat. *Huazhong argicultural university*.

Wang, Y. (2020). The research of residue eliminition and target animal safety for aditoprim unilateral and compound injection in swine. *Huazhong argicultural university*.
